# The Effects of Wubeizi Ointment on the Proliferation of Keloid-Derived Fibroblasts

**DOI:** 10.1007/s12013-014-0219-7

**Published:** 2014-10-25

**Authors:** Ji-cun Ding, Zhi-ming Tang, Xiao-xiang Zhai, Xiang-hui Chen, Jing-guo Li, Cui-xia Zhang

**Affiliations:** 1 Department of Plastic Surgery, Xuzhou Central Hospital, 199# South Jiefang Road, Xuzhou, 221009 Jiangsu China; 2 Department of dermatology, Xuzhou Hospital of Traditional Chinese Medicine, Xuzhou, 221003 Jiangsu China

**Keywords:** Wubeizi ointment, Keloid, Fibroblast, Primary cell culture, Cell proliferation

## Abstract

To evaluate the effectiveness of the Wubeizi (WBZ) ointment on keloid-derived fibroblasts. The primary cells of the keloid-derived fibroblasts were cultured and the effectiveness of the WBZ ointment at different concentrations was examined by MTT colorimetric methods on keloid-derived fibroblasts. The WBZ ointment showed inhibitory effects on proliferating the keloid-derived fibroblasts (*P* < 0.01)in a time- and dose-dependent manner. The proportion of cells in S stage was significantly higher in each of the WBZ ointment group than in the control group (*P*<0.01), and the proportion of G_2_ + M stage cells was significantly lower than that of control group, which was statistically significant (*P* < 0.01).The inhibitory effects of the S and G_2_ + M stage increased with higher drug concentrations (*P* < 0.05). Conclusion: The WBZ ointment can inhibit the proliferation of the keloid-derived fibroblasts in a time- and dose- dependent manner.

## Introduction

Keloids are characterized by the proliferation of dermal fibroblast and extracellular matrix (Type I and III) collagens, mucins and glycosaminoglycans. The Wubeizi (Rhus chinensis Mill.) (WBZ) ointment is an effective treatment of keloids [1]. Keloid-forming fibroblasts were used in this experiment to observe the efficacy of the Wubeizi ointment on the 4th to 8th generation fibroblastsat different drug concentrations.

## Materials and Methods

### Specimens

The keloid lesions were collected from patients for this study. The criteria for the keloids were as follows: at the phase of hyperplasia; the patients without connective tissue diseases or other illnesses that might affect metabolism, or other chronic heart, lung, liver, or kidney diseases; no steroid hormone drugs, penicillamine, or anticancer drugs used within the past 3 months; and no exposure to radiotherapy.

### Main Reagents

RPMI1640 medium (U.S.A HYCLONE, lot SH30011.01), dimethyl sulfoxide (Solon Ind. Pkwy. Solon, Ohio Irrilant Combustible Hygroscopic Lot: 1392B11), MTT (produced by Sigma, distributed by Jinan Aibo Biology Co., Ltd.), trypsin (CIBCO, distributed by Jinan Aibo Biology Co., Ltd.), and fetal bovine serum (Hangzhou Sijiqing Biological Engineering Materials Co., Ltd., batch number: 021009).

### Main Equipment

BB5060uv carbon dioxide incubator (Heraeus), XDS-LB inverted microscope (Chongqing Optoelectronic Company), double-row six well electric heated water bath (Shandong Medical Instrument Factory), XSZ-H ordinary optical microscope (Chongqing Optoelectronic Instrument Corporation), YXQ-SG46-280SA stainless steel portable pressure steam sterilizer (Shanghai Boxun Industries Co., Ltd. Medical Equipment Factory), BCM-1000A bio-clean bench (Sujing Group Suzhou Antai Air Technology Co., Ltd.), EL340i enzyme-linked immunoassay analyzer (U.S. Biotok), DU640 UV spectrophotometer (U.S.A Beckman), FA Calibor laser flow cytometer (U.S. BD).

### Drug Preparation

The drug composition and extraction are the same as the method for preparing Wubeizi ointment [1]. Solutions I and II were mixed to a concentration of 1 g/ml, and stored at −20 °C for future use. Before application, the solutions were diluted with pH 7.4 PBS to the set concentration. The concentrations were 1 g/ml for the high concentration group, 0.5 g/ml for the medium concentration group, and 0.25 g/ml for the low concentration group.

### Grouping

Keloid fibroblasts are divided into three groups of drug concentration, namely high dose, medium dose, low dose and the control group.

### Laboratory Experiment

#### Culture of Fibroblasts

The instruments were sterilized and balanced with a salt solution filtered and sterilized with filtration membranes. A keloid specimen was cut and washed in a salt solution to remove the blood. After the fat and connective tissues were removed, the specimen was placed in a petri dish containing culture medium, and cut into small cubes of 1 mm^3^ under microscope. After rinsing with the salt solution, the cubes were carefully transferred to a bottle coated with fetal calf serum using a pipette, and arranged properly with a glass rod. A small amount of culture solution was added before the flask was capped, and placed upside down in the CO_2_ incubator for 6–8 h. The bottle was then taken out and turned upright again, topped off with the culture solution, and placed in for further culture. The culture solution was replaced at an interval of 2–3 days.

When the cells had grown, the old solution was removed, and 2–3 ml of 0.25 % trypsin solution was added. The bottle was gently turned for 3–5 min, and the trypsin solution was discarded. Then, 10 ml of the medium was added, and a pipette was used to gently blow the solution against the cells on the bottle wall so that the cells were freed and formed a suspended single cell. The cells were counted using a cell counting chamber under microscope. The required cells were taken and divided into new flasks, filled with culture solution, and put into a 5 % CO_2_ incubator at 37 °C for a sterile culture. The generation was passaged every 2–3 days. The 4th to 8th generations’ cells at logarithmic growth phase were analyzed.

#### Keloid fibroblast Proliferation at Different Drug Concentrations Detected with MTT Colorimetric Assay

Fibroblasts of the 4th generations at the logarithmic growth phase were treated with trypsin before counting. The cell number was adjusted to 2 × 10^5^/ml, and the cells were seeded in 96-well plates, 150 μl in each well. A complete blank middle was used as the control group. Six duplicate wells were set in each group. Both groups were pre-incubated at 37 °C in a CO_2_ incubator for 24 h. The test drugs were filtered and the PH values adjusted with PBS, and prepared as three concentrations: 1 g/ml (high), 0.5 g/ml (medium), and 0.25 g/ml (low), with 50 μl in each well. After incubation at 37 °C in a CO_2_ incubator for 12, 24, and 36 h, the cells were incubated for another 4 h after adding 20 μl MTT to each well. After the incubation, the liquid was removed, and 150 μl/well of dimethyl sulfoxide was added. After shaking to ensure dissolution of the blue-purple formazan, the products were placed under a microplate reader at a wavelength of 490 nm to measure the OD values 1 h later. The mean OD values of the six wells at different concentrations were used to calculate the proliferation rate of the cells, as follows:

$${\text{Cell proliferation rate }} = {\text{drug group mean OD}}/{\text{blank control mean OD}} \times 1 0 0 \%$$

#### Measurement of the DNA Cycles of Keloid Fibroblasts at Different Concentrations of the Drugs

The cells were seeded in a 50 ml flask at 5 × 10^5^ cells/flask and cultured for 24 h. The original culture solution was discarded, and 1.5 ml of the culture solution and 0.5 ml of the Wubeizi ointment at different dose concentrations were added. A blank control group with 2 ml culture solution was established. After 24 h of incubation, the cells were treated in 0.25 % trypsin for collection. The cell count was adjusted to 1 × 10^6^/l, and placed in a water bath with 20ul of 1 mg/ml RNA enzyme at 37 °C for 30 min. PI 1 ml was then added for staining for 10 min at room temperature. The cells were collected by flow cytometry, with an excitation wavelength of 488 nm. The DNA level of each cell cycle was analyzed with the Modifit software.

### Statistical Analysis

Measurement data were expressed as mean and standard deviation ($\overline{x} \pm s$). The Bonferroni method was used to compare the Intergroup for normality and homogeneity; otherwise the Tamhane’s T_2_ method was used for variance test and pairwise comparisons. Data were processed with SPSS 11.0 for Windows. The homogeneity test of variance method (F test) was used in this study.

## Results

### Inhibitory Effect of Wubeizi Ointment Solution on Fibroblast Proliferation

As the results show, there were very significant differences in mean OD values between the medium and high dose groups and the control group (*P* < 0.01). With higher drug concentrations, the inhibitory effect on proliferation increased, with a significant difference between the high and low dose groups (*P* < 0.01). This suggests that Wubeizi ointment concentration inhibits the proliferation of fibroblasts. Twenty-four hours after adding the drugs, there were very significant differences between the high and medium dose groups and the control group (*P* < 0.01). However, there was no difference between the three dose groups (*P* > 0.05). Thirty-six hours after the administration, there were very significant differences in each dose group compared with the control (*P* < 0.01). However, there was no difference between the three dose groups (*P* > 0.05) Table 1.

**Table 1:** OD value at 490 nm wave length on 12 h, 24 h, and 36 h by MTT colorimetric method**(** $\overline{x} \pm s$ **)**

Groups	n	12 h		24 h		36 h	
		OD	%	OD	%	OD	%
Control group	6	0.701 ± 0.104	100	0.554 ± 0.130	100	0.413 ± 0.033	100
Low dose group	6	0.650 ± 0.076	92	0.465 ± 0.128	84	0.244 ± 0.098*	59
Medium dose group	6	0.451 ± 0.075*^△^	64	0.286 ± 0.037**	51	0.174 ± 0.055*	42
High dose group	6	0.364 ± 0.099*^△^	51	0.233 ± 0.041**	42	0.126 ± 0.018^*^	31

12 h F value = 36.48, *P* = 0.000 < 0.001.* *P* < 0.01 vs control group, ^**△**^ *P* < 0.01 vs low dose group. 24 h F value = 28.68, *P* = 0.000 < 0.001. *P* < 0.01 vs control group. 36 h F value = 49.41, *P* = 0.000<0.001. * *P* < 0.01 vs control group. by analysis of covariance

**Fig. 1:**
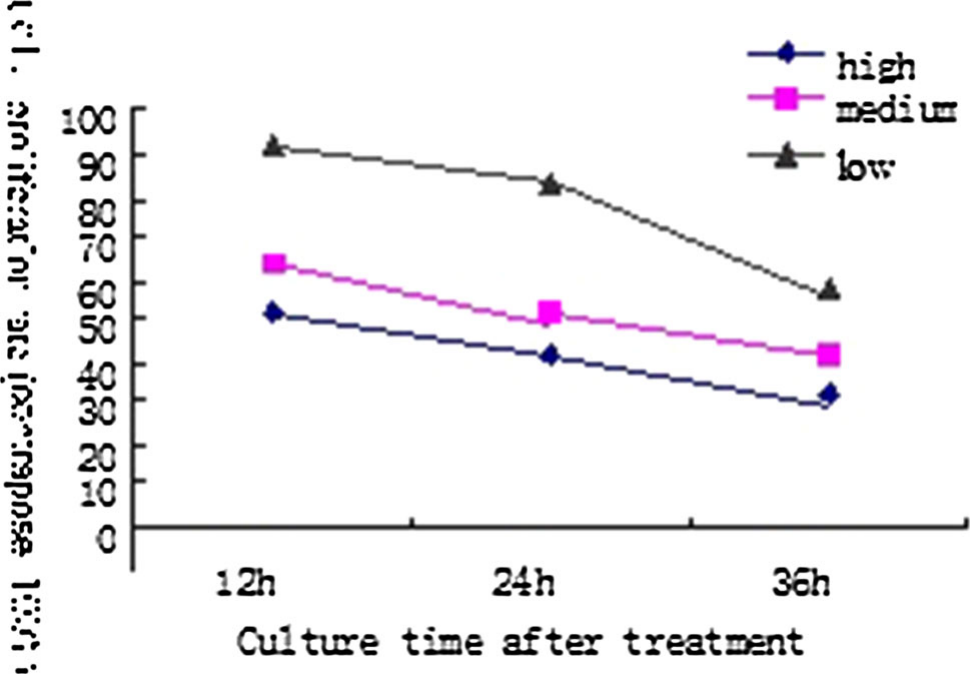
Inhibitory effects of different concentrations of WBZ ointment on the proliferation of keloid-derived fibroblasts

The proliferation rates at the three time points, suggested that at different doses, the ointment manifested inhibitory effects against the fibroblast, independent of time and dose, as shown in Fig. 1.

### Wubeizi Ointment Solution on the Cell Cycle of Keloid Fibroblasts

Compared to the control group, there was no difference in the percentage of G_0_ + G_1_ phase cells in the high dose group (*P* > 0.05), but there was a significant difference in the number of S and G_2_ + M cells (*P* < 0.01).

**Fig. 2:**
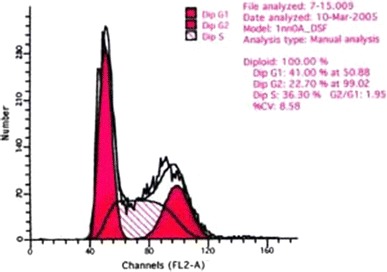
DNA cycle of keloid-derived fibroblasts in the control group

**Fig. 3:**
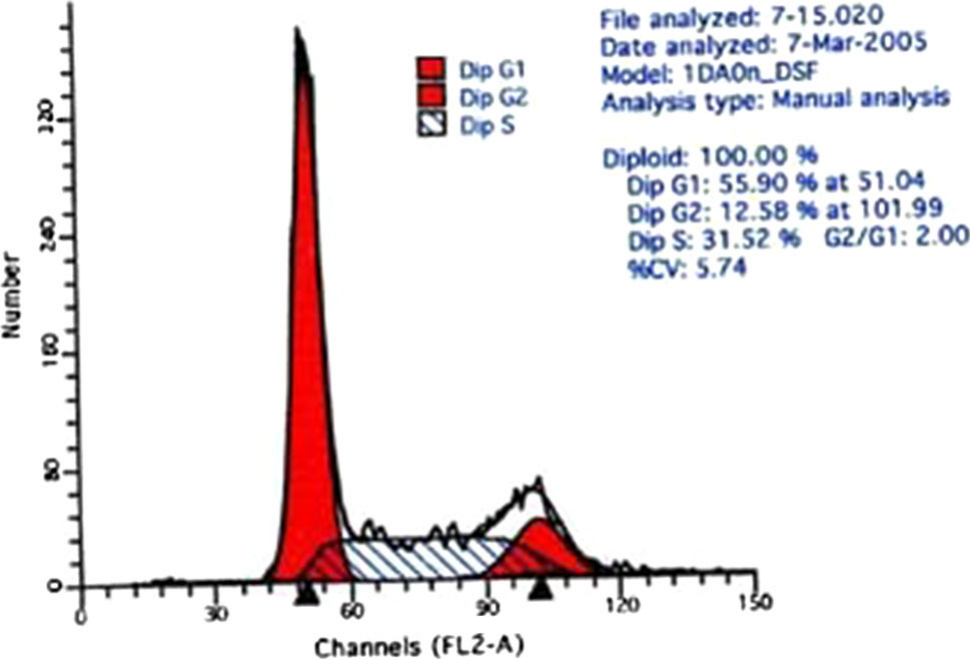
DNA cycle of keloid-derived fibroblasts in low dose group

**Fig. 4:**
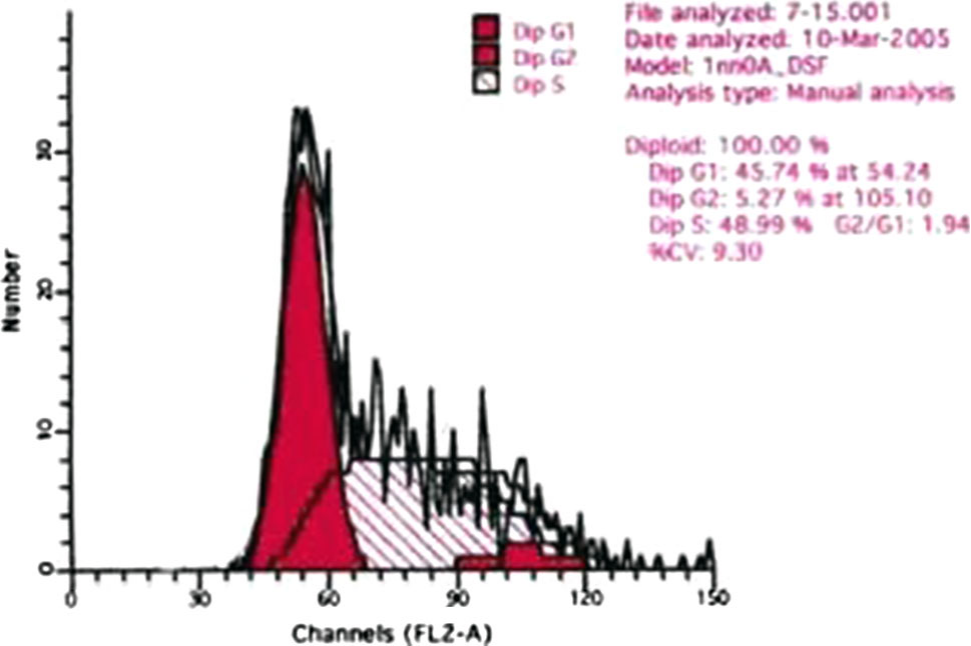
DNA cycle of keloid-derived fibroblasts in medium dose group

**Fig. 5:**
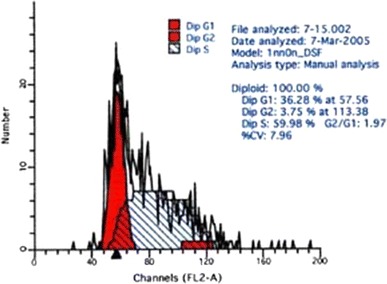
DNA cycle of keloid-derived fibroblasts in high dose group

Compared with the control group, there were very significant differences in the number of S and G_2_ + M phase cells in the medium dose group (*P* < 0.01), and in the number of G_2_ + M phase cells in the low dose group (P < 0.01). Compared with the low dose group, there were very significant differences in the number of S and G_2_ + M phase cells in the high dose group (*P* < 0.01), very significant differences in the number of S phase cells in the medium dose group (*P* < 0.01) and significant differences in the G_2_ + M phase cells in the medium dose group (*P* < 0.05). There was a very significant difference in the number of S phase cells between the high and the medium dose groups (*P* < 0.01) Table 2 and Figs. 2, 3, 4 and 5.

**Table 2:** Influence of WBZ ointment on keloid-derived fibroblasts DNA cycle(%) ($\overline{x} \pm s$)

Groups	*n*	G_0_ + G_1_	S	G_2_ + M
Control group	5	40.97 ± 4.47	37.32 ± 2.93	22.42 ± 5.07
Low dose group	5	58.16 ± 1.23**	33.91 ± 3.03	11.97 ± 1.96**
Medium dose group	5	46.52 ± 4.70^△^	48.85 ± 4.92**^△△^	6.01 ± 2.09**^△^
High dose group	5	36.07 ± 4.24^△△◊^	60.35 ± 5.75**^△△◊◊^	3.16 ± 0.49**^△△^

** *P* < 0.01 vs control group. ^△^ *P* < 0.05, ^△△^ *P* < 0.01 vs low dose group, and ^◇^ *P* < 0.05, ^◊◊^ *P* < 0.01 vs medium dose group

## Discussion

Keloids are formed as a result of abnormal overgrowth of scar tissue after the healing of skin wounds. Fibroblasts are primarily responsible for wound healing. The abnormal collagen activation, proliferation, synthesis, and differentiation in connection with fibroblasts in wound healing play an important role in the formation of keloids. Fibroblasts at the resting state are a component of skin connective tissue cells, often remaining in a low metabolic and inactive state. In the case of injury to the human skin, fibroblasts are activated to migrate along the established fiber network of the injured site under the chemokines effect. As they occupy the wound, the fibroblasts will proliferate, trigger the synthesis of collagen and other extracellular matrix, or induce wound contraction. Keloids are characterized by excessive production of collagen and other extracellular matrix [2], in which fibroblasts are primarily responsible for the formation of collagen fibers. The excessive proliferation to synthesize collagen should be a critical part in this process. Therefore, determining whether a therapeutic mechanism in treating keloids involves the inhibition of keloid fibroblasts proliferation, acceleration of fibroblasts apoptosis, or compromising their ability to synthesize collagen.

As shown in the results, all treatment groups at different doses present keloid fibroblasts inhibition to a certain extent, with significant differences between the high and medium dose groups versus the low dose group at 12 h. The results suggest that the inhibitory effect is independent of drug concentration and time.

The cell cycle assay of keloid fibroblasts in vitro showed significantly higher percentages of S phase cells in all treatment groups compared with the control group following the drug application (*P* < 0.01), and significantly lower percentages of G_2_ + M phase cells compared to the control group (*P* < 0.01).Thus, the blocking effect on the S phase keloid and the inhibitory effect on the G_2_ + M phase cells would intensify as the concentration increased (*P* < 0.05). These suggest that Wubeizi ointment increased the number of fibroblasts at the S phase, resulting in an S phase arrest and greatly reduced number of dividing cells, thus inhibiting the proliferation of keloid fibroblasts. In addition, the inhibition has a dose-dependent nature.

There will be a separate study on the Wubeizi ointment to investigate if it accelerates the apoptosis of fibroblasts and synthesize collagen.
